# Low-dose glucocorticoid improves progression-free survival of children with B cell acute lymphoblastic leukaemia following chimeric antigen receptor T-cell therapy

**DOI:** 10.3389/fimmu.2025.1604866

**Published:** 2025-10-29

**Authors:** Hui Zhang, Yuxuan Wang, Qi Ji, Qinyi Zhang, Chonglian Qiu, Saihu Huang, Xingqiang Dong, Jian Pan, Jun Lu, Zhenjiang Bai, Shaoyan Hu, Shuiyan Wu

**Affiliations:** ^1^ Pediatric Intensive Care Unit, Children’s Hospital of Soochow University, Suzhou, Jiangsu, China; ^2^ Hematology & Oncology, Children’s Hospital of Soochow University, Suzhou, Jiangsu, China; ^3^ Institute of Pediatric Research, Children’s Hospital of Soochow University, Suzhou, China; ^4^ Pediatric Hematology & Oncology Key Laboratory of Higher Education Institutions in Jiangsu Province, Suzhou, Jiangsu, China

**Keywords:** cytokine release syndrome, immune effector cell-associated neurotoxicity syndrome, glucocorticoid, chimeric antigen receptor T cell therapy, B cell acute lymphoblastic leukaemia

## Abstract

**Background:**

The prognostic impact of immunosuppressant therapies for cytokine release syndrome (CRS) and immune effector cell-associated neurotoxicity syndrome (ICANS), along with the outcomes and prognosis of children with relapsed/refractory B cell acute lymphoblastic leukaemia (B-ALL) undergoing chimeric antigen receptor (CAR) T-cell therapy, varies across populations. However, studies specifically focusing on these factors in the pediatric B-ALL population remain limited.

**Methods:**

We investigated the effects of immunosuppressants on outcome efficacy and prognosis in a retrospective cohort of 120 patients treated with CAR T-cell infusion at a single institution from March 2017 to August 2023. The 30-day complete response rate, progression-free survival (PFS), overall survival (OS), and event-free survival (EFS) were evaluated.

**Results:**

The median age of the patients was 8.0 years (range, 2.2–18.0 years). Following CAR T-cell therapy, 91.67% of patients developed CRS and 25.83% developed ICANS. At 1 month after CAR T-cell infusion, 70.83% of patients received tocilizumab (TCZ), 24.17% received ruxolitinib (RUX), and 50.83% received glucocorticoids (GC) for CRS or ICANS management. By day 30, 92.08% of patients achieved a complete response. The complete-response rates did not differ between the GC and non-GC, TCZ and non-TCZ, or RUX and non-RUX groups. The median follow-up time was 20.6 months (range, 4.26–38.82 months). OS, EFS, and PFS did not significantly differ between the RUX and non-RUX or TCZ and non-TCZ groups. However, patients receiving low-dose GC (≤ 8 mg kg^-^¹) exhibited better PFS than the non-GC group, with multivariable analysis demonstrating low-dose GC as an independent protective factor for PFS (hazard ratio, 0.45; 95% confidence interval, 0.21–0.96).

**Conclusions:**

In the context of CRS/ICANS management, low-dose GC independently confers long-term PFS benefits to pediatric B-ALL patients without compromising CAR T-cell activity when using appropriate GC, TCZ, or RUX regimens.

## Introduction

The management of cytokine release syndrome (CRS) and immune effector cell-associated neurotoxicity syndrome (ICANS) is pivotal for successful deployment of chimeric antigen receptor (CAR) T-cell therapy (1). Among the therapeutic strategies used to treat these conditions, glucocorticoid (GC), tocilizumab (TCZ), and ruxolitinib (RUX) exhibit varying degrees of efficacy and implications (2, 3). GCs are frequently utilized due to their potent anti-inflammatory properties, suppress the transcription of pro-inflammatory cytokines and reduce immune cell activity (4–6). Although GCs can effectively mitigate the symptoms of CRS and ICANS, their broad immunosuppressive effects pose significant limitations (7–9). Previous studies showed that GC influenced the number of CARs in five cases of relapsed B cell acute lymphoblastic leukaemia (B-ALL) (8) and 26 cases of relapsed and refractory B-ALL (9) following treatment with CAR T-cell therapy. In contrast, Gardner et al. demonstrated that treatment with GCs did not affect the anti-tumor activity of CAR T-cells (4). Data from 68 patients with relapsed B-ALL (both adults and children) from Beijing Boren Hospital in 2020 showed that GC use was not associated with the 30-day remission rate, nor did it affect the number and activity of CAR T-cells (10). Analysis of adults with lymphoma showed that use of GC affects patient outcomes, high-dose GC use is associated with worse progression-free survival (PFS), and early and long-term and high-dose GC use significantly shortens overall survival (OS) (7). A 2022 study of adult multiple myeloma revealed that the dose, initiation time, and duration of GC treatment did not affect the remission rate and long-term prognosis of patients treated with CAR T-cell therapy (11). More recently, a 2024 study of relapsed and refractory multiple myeloma showed that GC use did not affect the anti-tumour effects of CAR T-cells and did not affect the long-term prognosis of patients (6).

Furthermore, studies have shown inconsistent results across tumors, including in B-ALL, which may be related to a small number of patients or population-related effects. Notably, studies focusing on their application in pediatric B-ALL populations remain limited. This study was performed to investigate whether these treatments (GC, TCZ and RUX) influence the clinical outcome and safety profiles of children with B-ALL to improve the understanding of their efficacy and potential adverse effects. Because GC has shown the most inconsistent effects, we performed detailed investigation of this treatment.

## Methods

### Study design and population

This was a single-center, retrospective, observational cohort study of pediatric patients with relapsed/refractory (R/R) B-ALL who underwent CAR T-cell therapy targeting CD19 and/or CD20/CD22 at Children’s Hospital of Soochow University between 1 March 2017, and 1 August 2023 (ChiCTR2000032211). The exclusion criteria included prior CAR T-cell infusion at a different institution and enrolment in an ongoing clinical trial. For patients who received more than one CAR T-cell therapy dose at our center, only the results of the first round of administration were considered in this analysis.

### Data collection and assessment

We retrospectively collected the clinical data of patients, including their basic information (age, sex, weight, disease status, and relapse sites), pre-treatment intervention records (previous chemotherapy, immunotherapy, transplantation, and bone marrow blasts), CAR T-cell infusion (sources and targets of CAR T-cells), characteristics and management of CRS and ICANS (incidence, grading, remission, duration, and time to immunosuppressive interventions), and follow-up data.

The complete response (CR) status was evaluated by bone marrow smear, minimal residual disease detection using flow cytometry, quantitative polymerase chain reaction to detect the fusion gene, and, when feasible, deep sequencing for gene mutation associated with malignant clones. The treatment response of patients was defined as CR, partial response, and no response according to the Clinical Practice Guidelines for Acute Lymphoblastic Leukemia (ALL) (version 2.2021) published by the National Comprehensive Cancer Network (12). Adverse events, such as CRS and ICANS, were graded according to the guidelines of the American Society for Transplantation and Cell Therapy (13). The use of GC, RUX, and TCZ was documented, and the total dose of GC was calculated as the methylprednisolone equivalent dose (mg). Considering that the study subjects were children, the cumulative glucocorticoid dose was converted to mg/kg. To establish an objective, data-driven threshold, we analyzed the distribution of cumulative methylprednisolone doses (mg·kg^-^¹) in our cohort and selected the 75th percentile (upper quartile) as the cutoff value. This percentile corresponded to 8 mg·kg^-^¹, leading to the low-dose GC group (≤8 mg·kg^-^¹) and high-dose GC group (>8 mg·kg^-^¹). The effect of immunosuppressive therapy on treatment response at day 30 in children treated with CAR T-cell therapy was evaluated, along with their long-term outcomes (OS, event-free survival [EFS], and PFS).

### Cytokine detection assay

Cytokine detection was performed for all patients receiving CAR T-cell infusion depending on the clinical condition. Serum samples were evaluated using a human Th1/Th2/Th17 subset detection kit by flow cytometry (Flow Cytometry Fluorescence Method, Beckman Coulter, Brea, CA, USA) to detect soluble proteins. The cytokine profile included IL-2, TNF-α, IFN-γ, IL-4, IL-6, IL-10, and IL-17A.

### Study groups and outcome measures

To assess the influence of each intervention on efficacy outcomes, CAR T-cell-treated patients were stratified into two comparative groups. For GC analysis, patients were allocated either into a GC group (exposed to GC within 30 days of CAR T-cell infusion) or non-GC group (not exposed to GC within 30 days of CAR T-cell infusion). For TCZ and RUX analysis, the study population was analogously divided into TCZ and non-TCZ groups and RUX-treated and non-RUX-treated groups, respectively.

The primary outcome was PFS, calculated from the date of CAR T-cell infusion to the date of disease progression, last follow-up, or death from any cause (whichever occurred first). The secondary outcomes were as follows: 30-day complete response rate (CRR), OS (time from the date of CAR T-cell infusion to the date of last follow-up or death from any cause), and EFS (time from the date of CAR T-cell infusion to the first documented relapse, progression, treatment failure, treatment-related death, or last follow-up). The last follow-up date was 1 August 2024.

### Statistical analysis

The normality of continuous data was assessed using Kolmogorov–Smirnov test. If the data followed a normal distribution, results were expressed as the mean (± standard deviation), and comparisons between the two groups were analyzed using independent *t*-test. If the data did not follow a normal distribution, they were presented as medians with ranges, and comparisons between the two groups were analyzed using Mann–Whitney U test. Categorical data were presented as numbers and percentages of cases in each category. Chi-squared test was used to compare rates between the two groups, and Fisher exact probability test was used if necessary.

Kaplan–Meier curves were plotted for time-to-event endpoints: OS, EFS, and PFS. Log-rank test was used to calculate the differences between each subgroup. The preliminary results revealed no difference in prognosis between groups receiving TCZ or RUX and those who did not receive these treatments. However, comparison between the GC-treated and untreated groups revealed a difference in prognosis. To further investigate the relationship between GC administration and prognosis, the cumulative dose of GCs was divided into low- and high-dose groups according to the 75% quantile of the cumulative dose of GCs. Differences between these groups were analyzed using Kaplan–Meier curves.

To clarify the effect of GC on patient outcomes, univariate analysis was conducted. Variables with p < 0.2 were incorporated into the Cox regression model, which was used to identify independent impact factors influencing PFS. To determine the association between cofounding factors and PFS, hazard ratios (HRs) and 95% confidence intervals (CIs) were estimated with adjustment for independent impact factors. All data analyses were performed in SPSS (version 27.0) software (SPSS, Inc., Chicago, IL, USA) or R (version 4.4.1) software (The R Project for Statistical Computing, Vienna, Austria). A p < 0.05 was considered to indicate a statistically significant difference.

## Results

### Patients and treatment

The patient cohort was comprised of 120 patients, consisting of 81 males (67.50%) and 39 females (32.50%), with a median age of 8.0 years (range, 2.2–18.0 years). All patients received a lymphocyte depletion protocol with fludarabine (50 mg/m^2^/d×3d) and cyclophosphamide (500 mg/m^2^/d×3d) prior to CAR T-cell therapy. Nine pediatric patients underwent bone marrow transplantation prior to CAR T-cell therapy. Ninety-two (76.67%) received CD19+CD22 CAR T-cells, 19 (15.83%) received CD19 CAR T-cells, and 9 (7.50%) received CD19+CD22+CD20 CAR T-cells. Most (90.83%) CAR T-cells were autologous. The median infusion dose of CAR T-cells was 6.5 × 10^6^/kg. A total of 115 (95.83%) cases showed relapse, whereas 5 (4.17%) cases were primary refractory. Before receiving CAR T-cell therapy, 24 (20.00%) patients experienced a second or greater relapse. The primary site of relapse before the current CAR T-cell treatment was the bone marrow (80.87%). The median blast percentages in the bone marrow prior to lymphocyte depletion and pre-infusion were 20% and 6%, respectively. Seventy-two (60.00%) patients presented with minimal residual disease ≥10^-2^ in the bone marrow at relapse (**Table 1**).

**Table 1:** Baseline characteristics of the study population.

Characteristic	N=120
Gender, n (%)	
Male	81 (67.50)
Female	39 (32.50)
Median age at infusion (range, years)	8.0 (2.2, 18)
Median Weight (range, kg)	27 (11.5, 92)
Prior transplant, n (%)	
Yes	9 (7.50)
No	111 (92.50)
CAR-T target, n (%)	
CD19	19 (15.83)
CD19+CD22	92 (76.67)
CD19+CD22+CD20	9 (7.50%)
Origin of CAR-T cells, n (%)	
Autologous	109 (90.83%)
Allogenic	11 (9.17)
Infusion dose, median (range,10^6^/kg)	6.5 (2,15)
Disease status before CAR-T, n (%)	
Primary refractory	5 (4.17)
First relapse	91 (75.83)
Second or greater relapse	24 (20.00)
Relapse locations before CAR-T therapy, n (%)	
BM	93 (80.87)
BM+CNS	8 (6.95)
BM+Testicle	2 (1.74)
BM+CNS+Testicle	1 (0.87)
CNS	5 (4.35)
Testicle	5 (4.35)
Others	1 (0.87)
Disease status prior lymphodepletion	
BM blast, median (range, %)	20 (0,98)
≥50%blasts, n (%)	35 (29.17)
≥25% and<50%blasts, n (%)	17 (14.16)
≥5 and<25% blasts, n (%)	23 (19.17)
<5% blasts, n (%)	34 (28.33)
NA, n (%)	11 (9.17)
MRD evaluation prior lymphodepletion, n (%)	
MRD<10^-4^	15 (12.50)
MRD≥10^-4^ and <10^-3^	6 (5.00)
MRD≥10^-3^ and <10^-2^	14 (11.67)
MRD≥10^-2^	72 (60.00)
NA	13 (10.83)
Median percentage blast on BM preinfusion (range,%)	6 (1,42.3)

BM, bone Marrow; CNS: central nervous system; MRD, minimal residual disease; NA, not available.

Following administration of CAR T-cell therapy, 91.67% (110/120) of patients developed CRS (29.17% grade 1, 36.67% grade 2, and 25.83% grade 3 or greater) and 25.83% (31/120) of patients developed ICANS (13.33% grade 1, 9.17% grade 2, and 3.33% grade 3 or greater). **Table 2** shows an overview of the CRS/ICANS events and their management. In our study cohort, patients received CAR T-cells targeting three distinct antigen combinations: CD19+CD22, CD19 alone, and CD19+CD22+CD20. To account for potential heterogeneity in toxicity profiles arising from the different CAR constructs, we analyzed the incidence and severity of CRS and ICANS among patient groups infused with cells targeting these different antigens. The results demonstrated no significant differences in the toxicity profiles between the groups (**Supplementary Table 1**). During the first month following CAR T-cell infusion, 70.83% (85/120) patients received TCZ, 24.17% (29/120) patients received RUX, and 50.83% (61/120) patients received GC, with a median cumulative methylprednisolone dose of 4 mg/kg. The median time to CRS onset was 2 days, with a duration was 6 days, whereas ICANS onset occurred after 1 day and lasted for a median of 4.5 days. The median time from CAR T-cell infusion to the onset of CRS/ICANS requiring glucocorticoid (GC) intervention was 5 days (range, 1-11 days), with GC administration maintained for a median duration of 2 days (range, 1-18 days). Notably, the overall response rates were 93.45% for CRS and 93.56% for ICANS, demonstrating comparable efficacy of GC-based management across both toxicity syndromes (**Table 2**).

**Table 2:** The characteristics and management of CRS and ICANS.

Characteristic	N (%)
CRS events, n(%)	
Any grade	110 (91.67%)
grade 1	35 (29.17%)
grade 2	44 (36.67%)
grade ≥3	31 (25.83%)
ICANS events, n(%)	
Any grade	31 (25.83%)
grade 1	16 (13.33%)
grade 2	11 (9.17%)
grade ≥3	4 (3.33%)
CRS/ICANS management	
CRS onset, median (range, days)	2 (0,9)
CRS duration, median (range, days)	6 (1,19)
CRS resolution rate	95.45%
ICANS onset, median (range, days)	1 (0–8)
Duration of ICANS at grade 1 or 2, median (range, days)	4.5 (3–8)
ICANS resolution rate	93.56%
Time to GC initiation, median (range, days)	5 (1,11)
Duration of GC use after CAR-T therapy, median (range, days)	2 (1,18)
Immunosuppressive agents use within 30 days post-infusion, n(%)	110 (91.67%)
GC use	61 (50.83%)
TCZ use	85 (70.83%)
RUX use	29 (24.17%)

CRS, cytokine release syndrome; ICANS, immune effector cell-associated neurotoxicity syndrome; GC, glucocorticoids; TCZ, tocilizumab; RUX, ruxolitinib.

### Efficacy and safety outcomes

**Figure 1:**
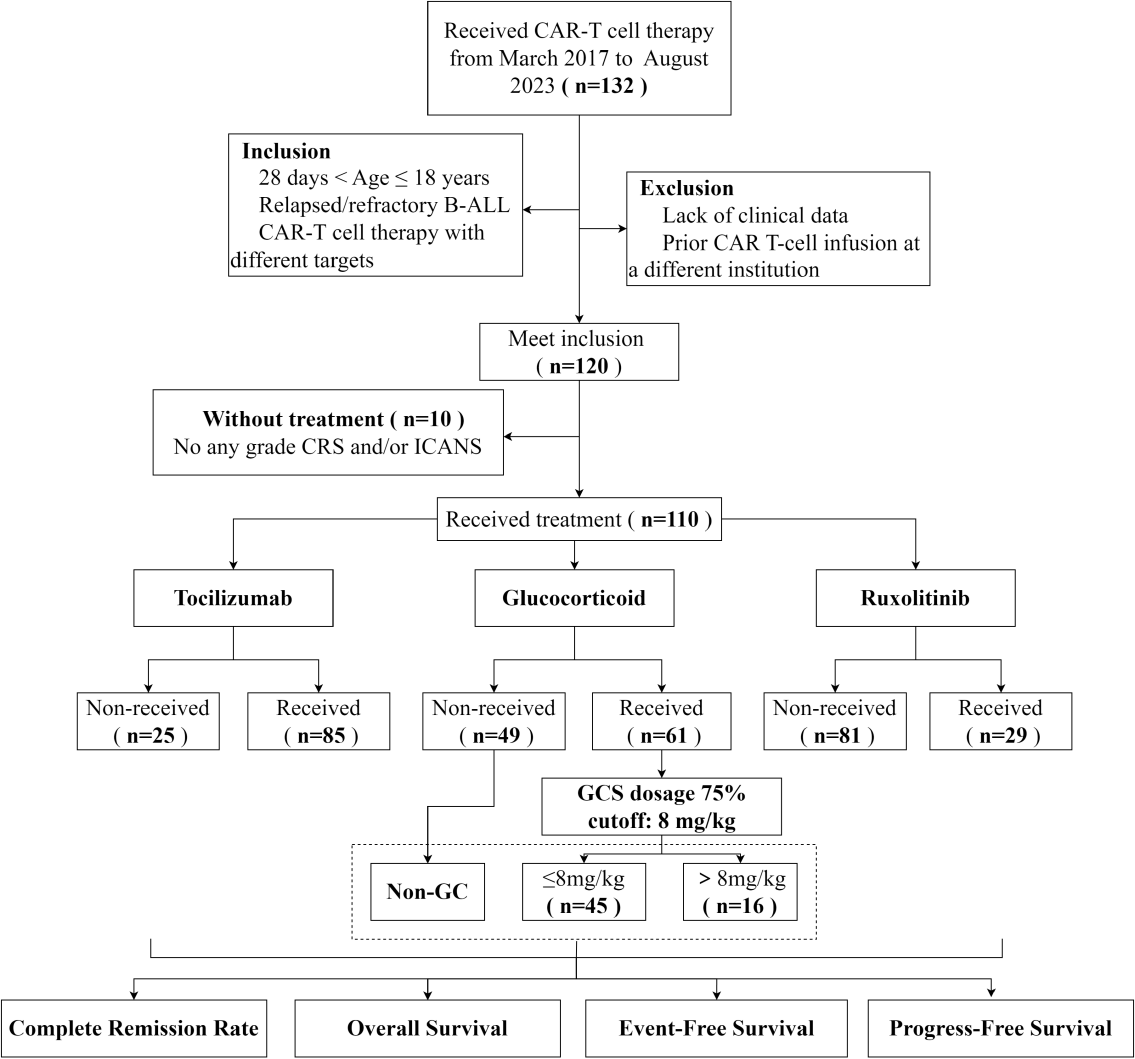
The flow chart of this study.

Among the 120 patients who underwent CAR T-cell therapy, 10 patients without CRS and/or ICANS were excluded; thus, 110 cases were selected for further analysis (**Figure 1**). The 30-day treatment response evaluation was available for 101 patients. Among them, 93 (92.08%) patients achieved a CR and 8 (7.92%) exhibited no response on day 30 post-CAR T-cell therapy. Additionally, patients were stratified into groups based on the use of immunosuppressive agents. In the non-GC group (n = 46), the CRR was 86.96% (40/46). For the GC group (n = 55), the CRR was 96.36% (53/55). Statistical analysis revealed no significant difference in the CRR between the non-GC and GC groups (p = 0.17). Furthermore, the CRR did not significantly differ for patients administered RUX and those not treated with RUX (p s= 0.14). Similar results were observed for the TCZ and non-TCZ groups (p = 0.189) (**Table 3**).

**Table 3:** Analysis between immunosuppressive agents and response to CAR-T infusion at day 30.

Response to CAR T cells	GC	Non-GC	TCZ	Non-TCZ	RUX	Non-RUX
CR	53	40	74	19	26	67
NR	2	6	4	4	0	8
*p*-value		0.17		0.14		0.189

The *p*-value was calculated using the Chi-square test. Note: CR, complete remission; NR, no response; GC, glucocorticoids; TCZ, tocilizumab; RUX, ruxolitinib.

At the end point of August 2024, the median duration of follow-up was 20.6 months (range, 4.26–38.82 months). The OS rates of the cohort at 1, 2, and 3 years were 80.7% ± 7.7%, 73.47% ± 8.94%, and 71.81% ± 9.32%, respectively. EFS rates at 1, 2, and 3 years were 69.93% ± 8.8%, 59.27% ± 9.9%, and 57.67% ± 10.13%, respectively. PFS rates were 71.30% ± 8.10%, 62.34% ± 8.82%, and 60.82% ± 8.99% at 1, 2, and 3 years, respectively (**Supplementary Figure 1**). Additionally, OS, EFS, and PFS analyses were conducted across these subgroups based on the use of GC, RUX and TCZ. The results showed no significant differences between the RUX and non-RUX subgroups in OS (p = 0.75), EFS (p = 0.88), and PFS (p = 0.51). Similar results were observed in the TCZ and non-TCZ subgroups. However, OS, EFS, and PFS clearly exhibited differing trends between the GC and non-GC groups, with the GC group exhibiting better outcomes than the non-GC group; further studies are needed to understand the reasons for these differences (**Supplementary Figure 2**).

**Figure 2:**
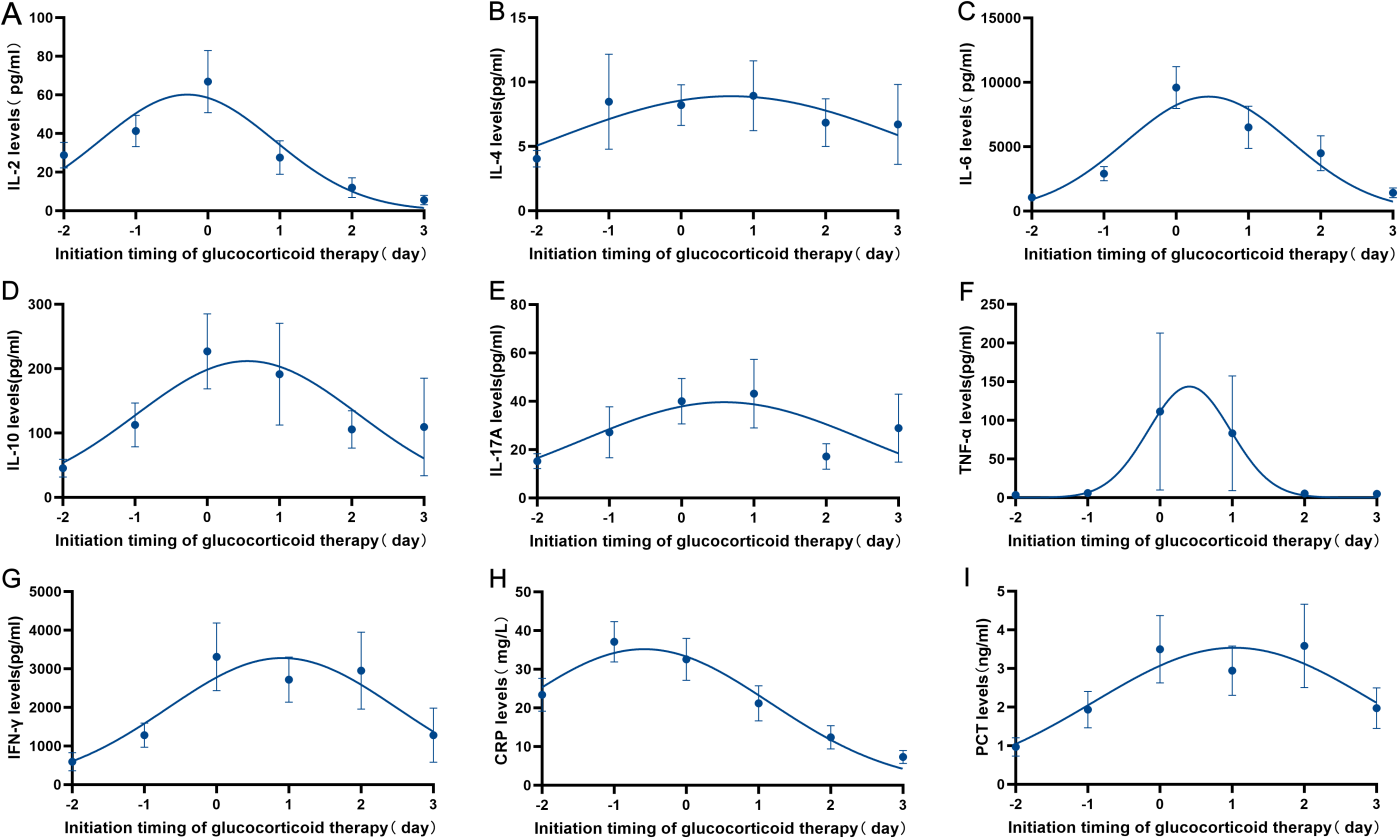
Dynamic monitoring of inflammatory markers (CRP and PCT) and the peripheral cytokine levels (IL-2, IL4, IL-6, IL-10, IL-17A, TNF-α and IFN-γ) before and after GC therapy was conducted in GC group (n=61). **(A–G)** Changes in cytokines (IL-2, IL-4, IL-6, IL-10, IL-17A, TNF-α and INF-γ) in patients with CRS/ICANS treated with glucocorticoids. **(H–I)** Changes in inflammatory markers (CRP and PCT) in patients with CRS/ICANS treated with glucocorticoids.

Subsequently, an in - depth analysis was carried out on the inflammatory markers (CRP and PCT) and the peripheral cytokine levels (IL-2, IL4, IL-6, IL-10, IL-17A, TNF-α and IFN-γ) after CAR-T cells infusion in both the GC group and the non - NGC group. The analysis results indicated that the levels of inflammatory markers in patients who received GC therapy were notably higher than those in the non-GC group (**Supplementary Figure 3**), which was in line with clinical observations. Furthermore, for patients undergoing GC treatment, a dynamic monitoring of the inflammatory markers (CRP and PCT) and the peripheral cytokine levels (IL-2, IL4, IL-6, IL-10, IL-17A, TNF-α and IFN-γ) was conducted both before and after the administration of GC in the GC group (n = 61). The findings clearly demonstrated a rapid decline in the levels of inflammatory markers and cytokines following the initiation of GC treatment (**Figure 2**).

### Impact of GC on the outcome of pediatric patients with B-ALL receiving CAR T-cell therapy

**Figure 3:**
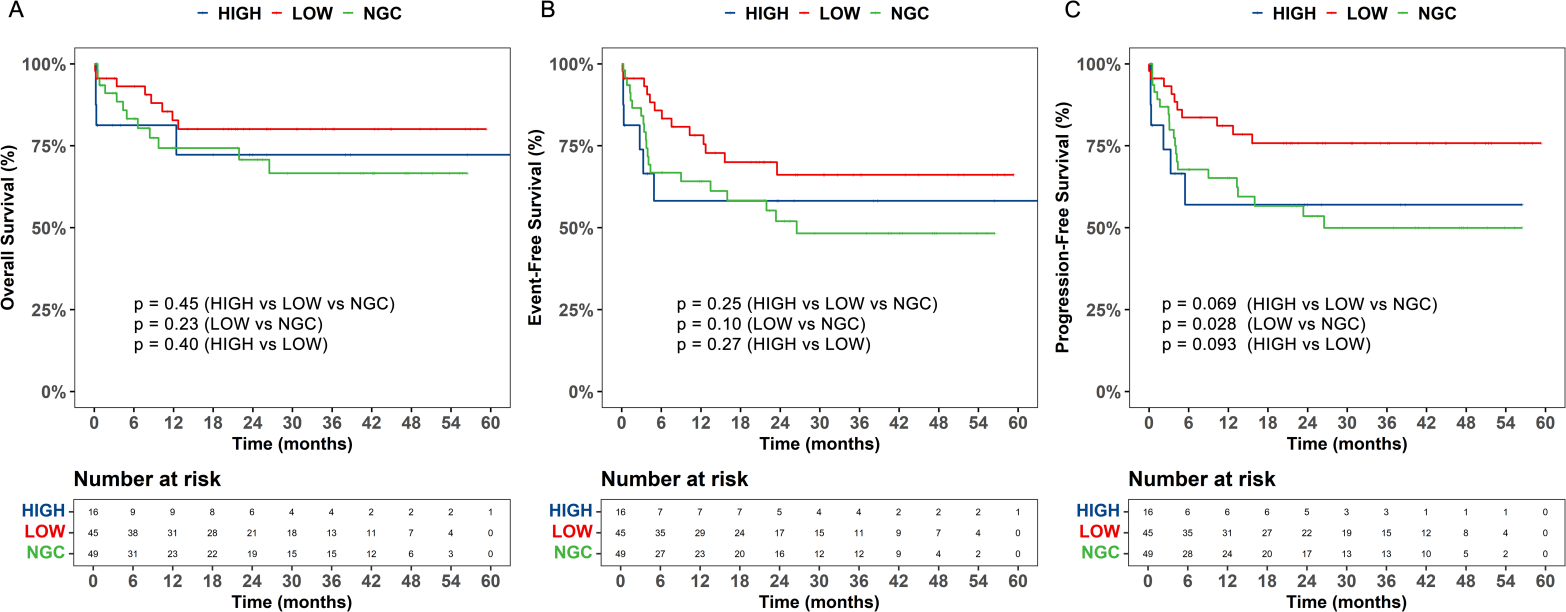
The impact of different dosages of glucocorticoids **(GC)** on overall survival (OS), event - free survival (EFS), and progression - free survival (PFS) of patients undergoing chimeric antigen receptor T - cell (CAR - T) therapy. **(A)** The overall survival (OS), **(B)** event-free survival (EFS), and **(C)** progression-free survival (PFS) were analyzed based on dose-based grouping of glucocorticoids use. LOW, low-dose glucocorticoid, HIGH, high-dose glucocorticoid, NGC, non-glucocorticoid.

To further examine the effect of GC on efficacy outcomes and prognosis following CAR T-cell therapy in pediatric patients, patients were divided into high-dose GC (>8 mg/kg), low-dose GC (≤8 mg/kg), and non-GC groups. The 30-day response rates after CAR T-cell therapy across the different GC dosage groups did not significantly differ (p = 0.181) (**Table 4**). Next, the impact of the onset and duration of GC on the 30-day response was assessed. Patients were divided into early and late initiation groups based on the median time. No significant differences in 30-day response rates were observed between these groups (p = 0.488). Similarly, patients were divided into short- and long-term treatment groups based on the median duration of GC therapy. The results showed that the duration of treatment did not affect the 30-day response (p = 0.393) (**Table 4**). Further exploration of the impact of GC dosage on OS, EFS, and PFS revealed that the low-dose GC group (≤8 mg/kg) had better OS, PFS, and EFS than did the high-dose GC (>8 mg/kg) and non-GC groups. The low-dose GC group (n = 45) showed a significantly longer PFS compared to the non-GC group (n = 49, p = 0.028), but the differences for OS (p = 0.23) and EFS (p = 0.10) were not significant between these two groups (**Figure 3**). To avoid confounding effects from RUX, the subgroup of patients receiving GC and TCZ but not RUX was further explored. The patients treated with a combination of TCZ and GC were divided into high- and low-dose GC groups. The low-dose GC group had a longer PFS compared to the high-dose GC group (**Supplementary Figure 4**). To determine the reason for this difference, we analyzed inflammatory markers and cytokine levels. IL-2, IL-6, IL-10, IFN-γ, CRP, and PCT were decreased in both the low- and high-dose GC groups, whereas IL-4 and IL-17A were increased in the high-dose GC group, and TNF-α was consistently very low in the high-dose GC group (**Supplementary Figure 5**). We also explored the importance of the timing and duration of GC treatment in prognosis. The results revealed no significant associations between the time of GC initiation (<5 vs. ≥5 days) or duration of GC treatment (≤2 vs. >2 days) in terms of OS, EFS, and PFS (**Supplementary Figure 6**).

**Table 4:** Association between the timing, duration, and dosage of GC and response to CAR-T infusion at day 30.

Response to CAR T cells	onset of GC use		Duration of GC use		dosage of GC use		
	<5 days	≥5 days	≤2 days	>2 days	0mg/kg	≦8mg/kg	>8mg/kg
CR	27	26	30	23	40	41	12
NR	0	2	0	2	6	1	1
*p*-value		0.488		0.393			0.181

The *p*-value was calculated using the Chi-square test. Note: CR, complete remission; NR, no response; GC, glucocorticoids; TCZ, tocilizumab; RUX, ruxolitinib.

### Multivariable model

The findings demonstrate that the PFS of patients with R/R B-ALL who experienced CRS/ICANS following CAR-T therapy was significantly better in the low-dose GC group (n = 46) than in the non-GC group (n = 49). To further examine the importance of GC in PFS, a Cox regression model was constructed to analyze confounding factors associated with PFS. In the univariate analysis, sex (p = 0.026, HR = 2.07, 95% CI 1.09–3.93), low-dose GC (≤ 8 mg kg^-^¹) (p = 0.032, HR = 0.44, 95% CI 0.20–0.93) and a pre-CAR-T conditioning bone-marrow smear with blasts ≥ 5% (p = 0.080) all met the preset inclusion criterion of p < 0.20 for the multivariable model. Multivariate Cox analysis confirmed that being female (p = 0.010, HR = 2.78; 95% CI 1.28–6.04) and pre-conditioning bone-marrow blasts ≥ 5% (p = 0.029, HR = 3.19; 95% CI 1.13–9.03) were independent adverse prognostic factors, whereas low-dose GC remained a protective factor for PFS (p = 0.017, HR = 0.37; 95% CI 0.16–0.84) (**Table 5**).

**Table 5:** Univariable and multivariate Cox model assessing the correlation between individual clinically-relevant covariates and progression-free survival (PFS).

Variables	Univariate analysis		Multivariate analysis	
	*P*	HR (95%CI)	*P*	HR (95%CI)
Weight				
≤27 kg		1.00 (Reference)		
>27 kg	0.801	0.92 (0.49-1.74)		
Sex				
Male		1.00 (Reference)		1.00 (Reference)
Female	0.026	2.07 (1.09-3.93)	0.010	2.78 (1.28-6.04)
Bone marrow blast percentage				
<5%		1.00 (Reference)		
≥5%	0.080	2.02(0.92-4.41)	0.029	3.19(1.13-9.03)
Age at CAR-T therapy				
≤8 years		1.00 (Reference)		1.00 (Reference)
>8 years	0.187	0.65 (0.34-1.23)	0.703	0.86 (0.39-1.89)
CAR-T target antigen				
CD19		1.00 (Reference)		
CD19+CD22	0.50	0.64(0.17-2.37)		
CD19+CD22+CD20	0.95	0.97(0.34-2.74)		
Transplantation preinfusion				
No		1.00 (Reference)		
Yes	0.375	1.71 (0.52-5.58)		
Disease status preinfusion				
Primary refractory		1.00 (Reference)		
First relapse	0.488	2.02 (0.28-14.89)		
Second or greater relapse	0.360	2.63 (0.33-20.77)		
GC dosage				
0 mg/kg		1.00 (Reference)		1.00 (Reference)
≤8 mg/kg	0.032	0.44 (0.20-0.93)	0.017	0.37 (0.16-0.84)
GC onset				
≤4 days		1.00 (Reference)		
>4 days	0.693	1.21 (0.47-3.14)		
GC duration				
1–2 days		1.00 (Reference)		
>2 days	0.910	1.06 (0.41-2.74)		
RUX use				
No		1.00 (Reference)		
Yes	0.643	0.83 (0.38-1.83)		
TCZ use				
No		1.00 (Reference)		
Yes	0.712	1.18 (0.49-2.85)		
CRS occurrence				
No		1.00 (Reference)		
Yes	0.848	0.90 (0.32-2.55)		
CRS grade				
Grade 1		1.00 (Reference)		
Grade 2	0.614	1.24 (0.54-2.83)		
≥Grade 3	0.388	1.49 (0.60-3.66)		
ICANS occurrence				
No		1.00 (Reference)		
Yes	0.552	1.23 (0.62-2.44)		
ICANS grade				
Grade 1		1.00 (Reference)		
Grade 2	0.780	0.84 (0.24-2.88)		
≥Grade 3	0.503	0.49 (0.06-3.98)		

Univariate and multivariate analyses were performed using the Cox proportional hazards regression model; CR, complete remission; NR, no response; GC, glucocorticoids; TCZ, tocilizumab; RUX, ruxolitinib; CRS, cytokine release syndrome; ICANS, immune effector cell-associated neurotoxicity syndrome.

## Discussion

CAR T-cell therapy is an effective treatment for refractory and/or relapsed B-ALL. However, treatment-related toxicities, such as CRS and ICANS, have hindered its widespread use. In our cohort, the incidence of CRS was as high as 91.67%, exceeding values determined in previous studies (14–16). In contrast, the incidence of ICANS was only 25.83%, which is lower than previously reported (14, 15, 17). These disparities may be related to differences in the type of CAR T-cells employed. At present, GC, TCZ, and RUX are primarily used to control CRS and ICANS. In this study, over 50% of children were treated with GC within 1 month of CAR T-cell therapy. The levels of inflammatory markers and cytokines decreased rapidly after GC intervention, which was similar to the rapid and effective anti-inflammatory effect of GC in severe cases of CRS reported in previous studies (5). However, treatment with GC shows different effects across tumors (4, 7–11). At present, no studies have focused on the pediatric B-ALL population. In this study, 120 children with R/R B-ALL were treated with CAR T-cells, making this study the largest population analysis of pediatric B-ALL, with the aim of revealing the effect of immunosuppressant therapy on the efficacy and prognosis on these patients.

Nearly all published reports detailing the activity of CD19 CAR in B-ALL have focused on CR rates at 1 month, which occur in approximately 60–100% of patients (14, 15, 18–20). In our cohort, 92.08% of patients achieved CR at day 30, which agrees with previously reported data (21). In addition, analysis of the effect of each immunosuppressant on the 30-day remission rate of patients who experienced CRS/ICANS showed that GC, TCZ, and RUX do not affect the 30-day remission rate, which is consistent with reported results (6).

The OS and EFS of our population were better than those in previously reported data (14, 18). In the present study, in the 76.67% of patients who received (CD19+CD22) CAR T-cell infusion, the 1-year OS and EFS were higher than those previously reported for dual-target CAR T-cell therapy (22). Further analysis of the effect of immunosuppressant therapy on long-term prognoses showed a trend towards better outcomes in the GC group than in the non-GC group. Notably, marked attenuation of pro-inflammatory cytokines was observed in patients treated with GC, including in the levels of IL-2, IL-4, IL-6, IL-10, IL-17A, IFN-γ, and TNF-α. Previous studies showed that pro-inflammatory cytokines, such as IL-6, TNF-α, and IFN-γ, are markers of disease severity and are associated with poor outcomes (23). The serum levels of inflammatory markers were also assessed, and significant decreases in CRP and PCT levels was observed following GC administration (24).

Use of GC is currently controversial, and the lack of a standardized dosage makes clinicians reluctant to use GC. In this study, we divided patients receiving GC into low- and high-dose groups according to the 75% quantile of the cumulative dose of GCs. Notably, compared with the non-GC group, the low-dose GC group demonstrated a significant improvement in PFS. Decreased levels of IL-2, IL-6, IL-10, IFN-γ, CRP, and PCT were observed in both low- and high-dose GC groups, but IL-4 and IL-17A were increased in the high-dose GC group, and TNF-α consistently showed a very low level in the high-dose GC group. IL-4 and IL-17A may be involved in ALL progression by promoting the proliferation of leukaemia cells and inhibiting apoptosis, and can also indirectly affect disease progression by modulating the tumor microenvironment (25, 26). However, TNF-α inhibits disease progression by inducing apoptosis in leukaemia cells (27). Together, these results suggest that low-dose GC intervention can control the inflammatory storm in the short-term and has potential benefits for long-term survival by maintaining a reasonable immune balance and supporting sustained clearance of CAR T-cells. In contrast, high-dose GC may promote tumor progression by impairing CAR T-cells. Furthermore, high doses impair adrenal function, suppress muscle regeneration, and exacerbate loss of body mass (28), leading to poor prognosis.

This study had some limitations. First, this study was a retrospective analysis, and some children underwent concomitant treatment with GC with other immunosuppressants (TCZ and RUX), making it difficult to exclude the effects of drug-drug interactions. Second, because dynamic copy number monitoring data on CAR T-cells were lacking, the relationship between CAR T-cell therapy and clinical efficacy could not be accurately evaluated.

## Conclusions

Taken together, our findings indicate that among patients who experienced CRS/ICANS, immunosuppressants alleviate toxicity without compromising efficacy. Specifically, low-dose GC independently improves PFS in children with R/R B-ALL following CAR T-cell therapy within this cohort.

## Data Availability

The original contributions presented in the study are included in the article/**Supplementary Material**. Further inquiries can be directed to the corresponding author/s.
