# Supplementary figures and images for: Low-dose glucocorticoid improves progression-free survival of children with B cell acute lymphoblastic leukaemia following chimeric antigen receptor T-cell therapy
Source: Front Immunol. 2025 Oct 29;16:1604866. doi: 10.3389/fimmu.2025.1604866 (PMC12605194; doi:10.3389/fimmu.2025.1604866)

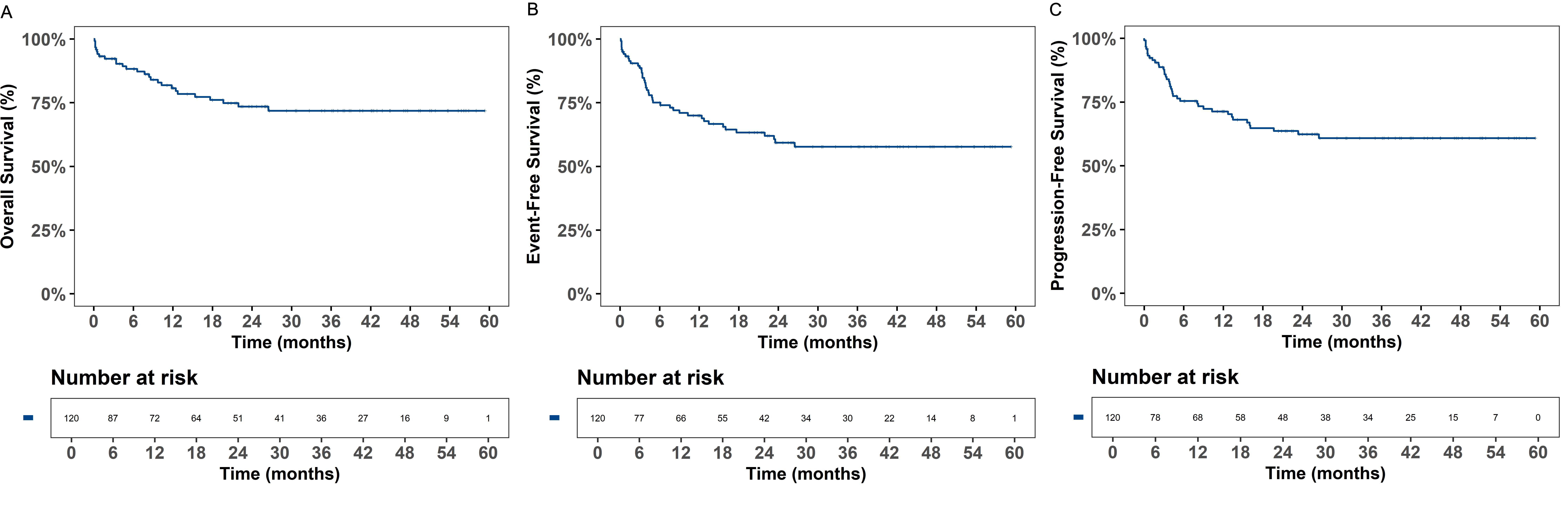

Supplement: Supplementary file 1 [file Image1.tif]

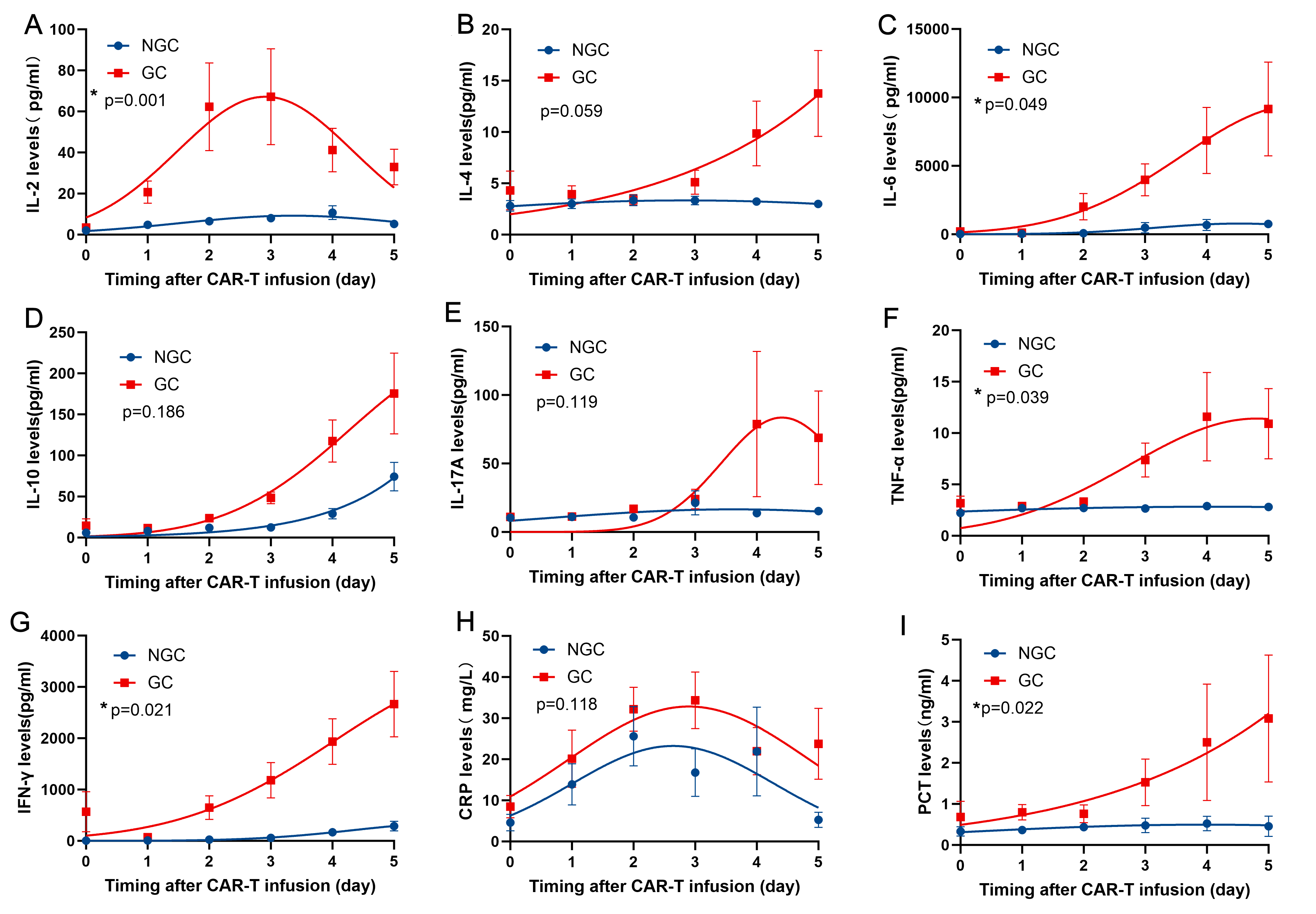

Supplement: Supplementary file 3 [file Image3.tif]

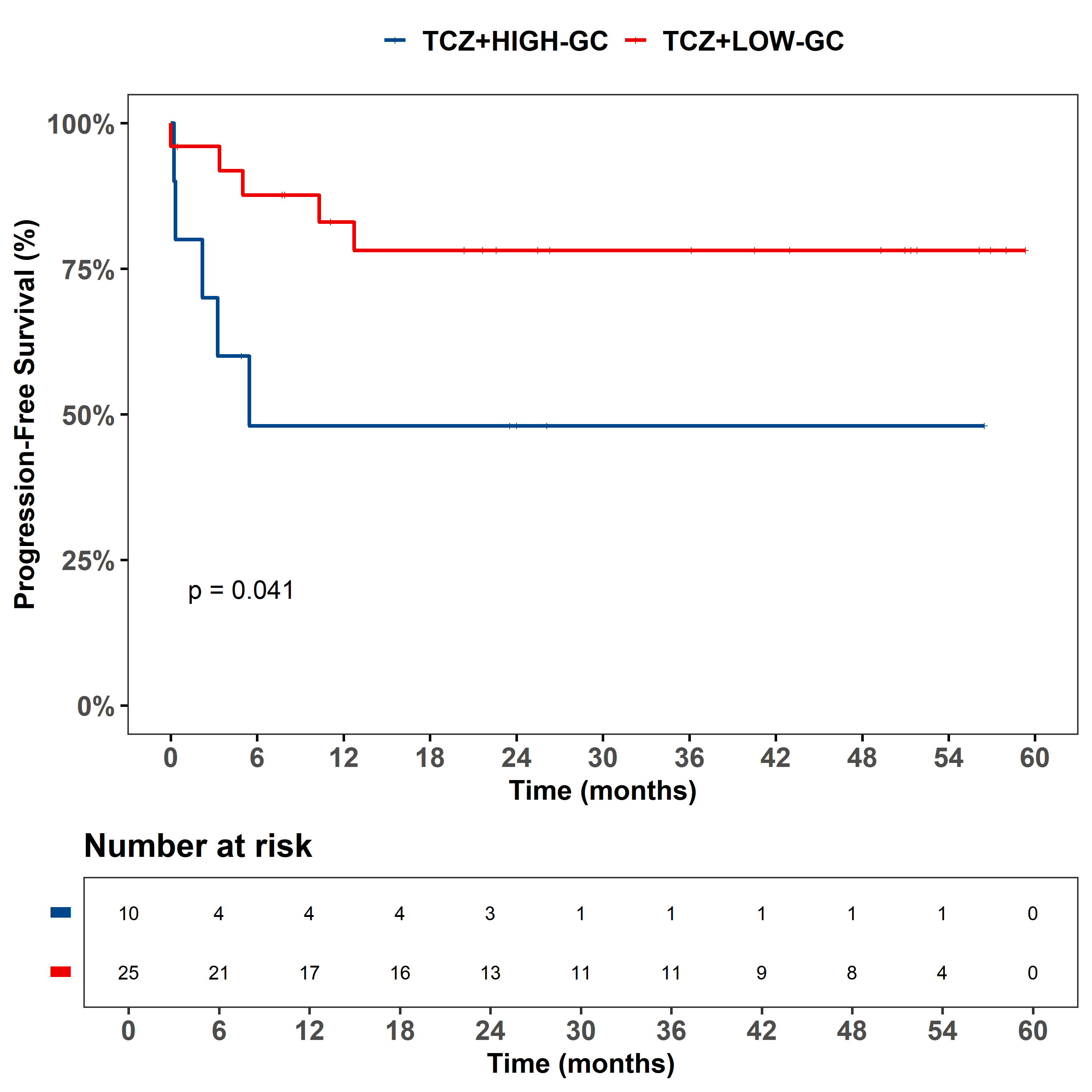

Supplement: Supplementary file 4 [file Image4.tif]

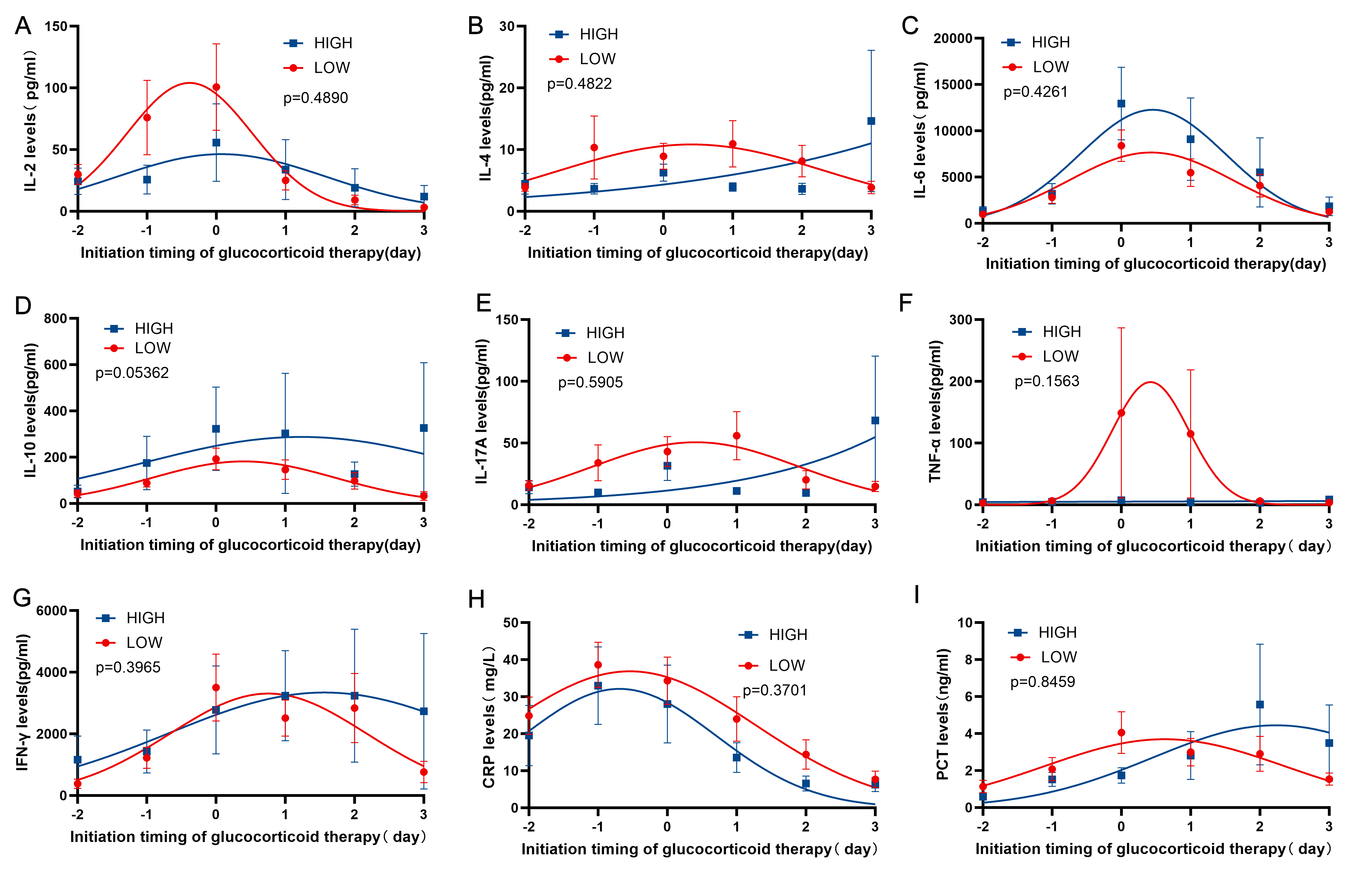

Supplement: Supplementary file 5 [file Image5.tif]

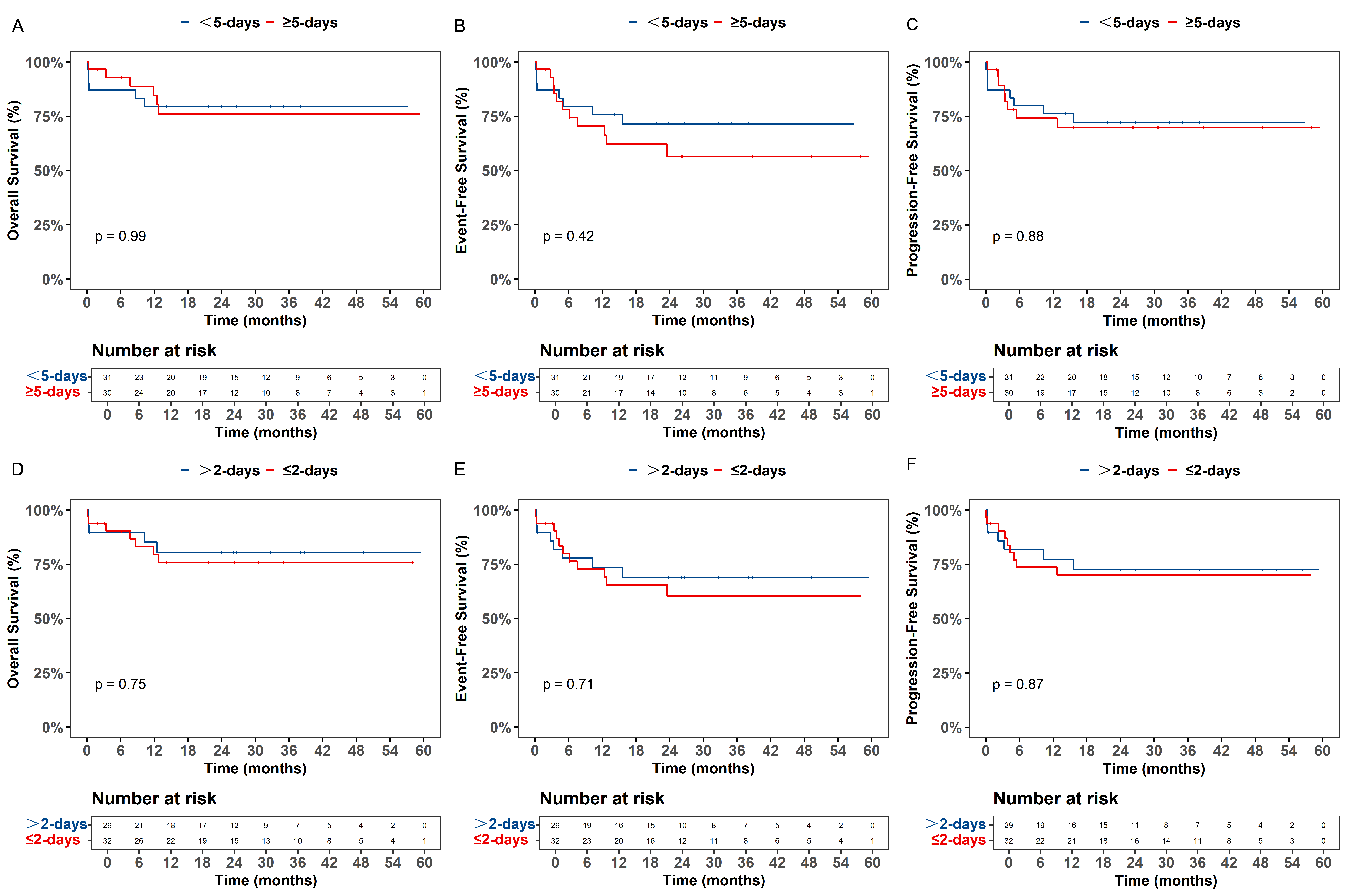

Supplement: Supplementary file 6 [file Image6.tif]
