# Supplementary material for: Low-dose glucocorticoid improves progression-free survival of children with B cell acute lymphoblastic leukaemia following chimeric antigen receptor T-cell therapy
Source: Front Immunol. 2025 Oct 29;16:1604866. doi: 10.3389/fimmu.2025.1604866 (PMC12605194; doi:10.3389/fimmu.2025.1604866)
Supplement: Supplementary file 7 [file Table1.docx]

**Supplemental Table1.Incidence and grading of cytokine release syndrome (CRS) and immune effector cell–associated neurotoxicity syndrome (ICANS) stratified by CAR target antigen**

|  | CD19（n=19） | CD19+CD22（n=92） | CD19+CD22+CD20（n=9） | *p*-value |
| --- | --- | --- | --- | --- |
| CRS events, n |  |  |  |  |
| Any grade | 16 | 85 | 9 | 0.322 |
| grade 1 | 6 | 26 | 4 |  |
| grade 2 | 5 | 37 | 2 |  |
| grade ≥3 | 5 | 22 | 3 | 0.706 |
| ICANS events，n |  |  |  |  |
| Any grade | 6 | 22 | 3 | 0.68 |
| grade 1 | 2 | 12 | 2 |  |
| grade 2 | 3 | 8 | 0 |  |
| grade ≥3 | 1 | 2 | 1 | 0.515 |
